# Health concerns of intensive care survivors and research participation willingness: A multicentre survey^☆^

**DOI:** 10.1016/j.ccrj.2024.04.001

**Published:** 2024-06-21

**Authors:** Reyas Aboobacker Kaniyamparambil, Charlotte Goldsmith, Nicolas Demasi, Brad Wibrow, Prakkash ParangiAnanthan, Adrian Regli, Matt Anstey, Susan Pellicano, Anne Marie Palermo, Sarah Van Der Laan, Edward Litton

**Affiliations:** a Intensive Care Unit, Fiona Stanley Hospital, Perth 6150, WA, Australia; b Joondalup Health Campus, Perth, 6027, Australia; c Intensive Care Unit, Sir Charles Gairdner Hospital, Perth 6009, WA, Australia; d Intensive Care Unit, Royal Perth Hospital, Perth 6000, WA, Australia; e St John of God Hospital Murdoch, Perth 6150, WA, Australia; f School of Medicine, University of Western Australia, 6009, WA, Australia

**Keywords:** Intensive care, Critical illness, Anaemia, Clinical trials, Patient reported outcome measures, Iron

## Abstract

**Objective:**

To describe the relative importance of health concerns reported by survivors of critical illness treated in the intensive care unit (ICU), their estimate of time to achieve recovery, and their reported randomised clinical trial participation willingness.

**Design:**

A multicentre survey.

**Setting:**

Six Australian ICUs.

**Participants:**

Adult patients who had received mechanical ventilation, vasopressor support or renal replacement therapy for more than 24 h were likely to be discharged from ICU within 24 h.

**Interventions:**

Survey administration was verbal and occurred in the ICU.

**Main outcome measures:**

A numeric rating of eight ICU survivor-related health concerns developed with consumer input (disability requiring ongoing care, prolonged hospitalisation, repeated hospitalisation, impaired activity level, pain, low mood, inability to return home, and dying). Zero indicated no concern and ten extreme concern. Respondents were also asked to estimate their expected recovery time and their willingness to participate in a randomised clinical trial.

**Results:**

Of 584 eligible participants, 286 (49.0%) respondents had a mean age of 62.3 years (standard deviation (SD) 14.8) and 178 (62.2%) were male. The median ICU length of stay at the time of survey was 4 days (interquartile range (IQR) 3–7). Respondents reported high levels of concern for all health outcomes with the highest median scores being for survival with severe disability and requirement for ongoing care scoring 8 (IQR 3–10), and never being able to return home needing assisted living or a nursing home scoring 8 (IQR 1–10). The median expected recovery time was 23 days (IQR 10–33). Higher concerns were associated with an increased likelihood of trial participation willingness.

**Conclusion:**

Survivors reported high and varied health concerns of which severe disability requiring care and inability to return home were the highest. Respondents anticipated a relatively short recovery.

## 1 Introduction

As mortality for patients treated in an intensive care unit (ICU) improves, outcomes beyond survival are increasingly recognised as important.^1^ However, relatively few studies have described outcome priorities reported by ICU survivors and such studies often describe patients’ perspective long after the ICU discharge.^2^ A greater understanding of the relative importance of different health outcomes to patients whilst in ICU, and the time period over which they may expect recovery to occur, can help inform clinicians and researchers in aligning therapies, discussing recovery expectations, and designing clinical trials consistent with the health outcomes that matter most to patients.^3^

Therefore, the primary aim of this study was to assess patient-reported health outcome concerns amongst survivors of critical illness during their intensive care admission and the timeframe over which patients expected their health to improve. A secondary aim was to evaluate whether differences in health outcome concerns were associated with a theoretical willingness to participate in randomised clinical trials (RCTs) aimed at improving recovery after ICU.

## 2 Methods

This was a multicentre survey of patients being treated in the ICU. Human Research Ethics Committee approval was obtained at all study sites prior to participant recruitment (St John of God Health Care #1778, Ramsay Health #2114W, South Metropolitan Health Service #4296).

### 2.1 Setting and participants

The study was conducted in the ICUs of six hospitals in Perth, Western Australia. Adult patients who had received mechanical ventilation, vasopressor support or renal replacement therapy for more than 24 h and had been identified as suitable for ICU discharge or considered by the treating clinician as likely to be discharged in the next 24 h, were eligible to participate. Patients who lacked the capacity to provide consent, who did not speak English, or for whom the treating clinician was not committed to active treatment were excluded.

### 2.2 Survey development

The survey questions were developed with input from consumers and in accordance with the Patient Reported Outcome Measures Information System (PROMIS) domains and included eight closed questions for which respondents were asked to rate their concern with respect to the outcome from zero being no concern to ten being extremely concerned.^4^ The eight outcomes were:

1. Surviving but being severely disabled and requiring ongoing care
2. Needing a long time in hospital to recover
3. Requiring repeated trips to hospital after discharge
4. Not being able to return to previous levels of activity
5. Uncontrolled pain
6. Ongoing anxiety and or low mood
7. Never being able to return home, needing assisted living or nursing home
8. Dying

Respondents were asked one opened-ended question of whether they had any other health concern. These were grouped into physical, mental/cognitive, social, and disease-orientated. Predicted time to improvement was assessed by first asking respondents’ health score on the survey day based on the visual analogue scale of the EuroQol EQ-5D where zero is the worst health imaginable and 100 is the best health imaginable.^5^ Then, respondents were asked to estimate their expected best health score once ICU recovery was complete, and finally asking their estimated time to achieve that recovery. Two planned ICU survivor recovery RCTs (intravenous iron and erythropoietin for anaemia, and anabolic steroids for prolonged ventilation) were explained to respondents and used to assess trial acceptability, alignment with health outcome concerns and theoretical willingness to participate (trial registration ACTRN12621000595819 and ACTRN12623000729628).

Face and content validity were assessed by piloting the survey on 10 ICU patients and comparing answers to the open-ended question of ‘*any other health concerns?*’ with the closed health domain questions using COSMIN methodology.^6^

### 2.3 Survey administration and addressing bias

Site investigators administered the survey face to face by approaching eligible patients in the ICU, gaining consent for participation, then reading out each question and recording the answers in real time. Clinical data were extracted concurrently from the medical record. Strategies to reduce bias included multicentre participation with broad eligibility criteria (sampling bias), enrolment of consecutive patients (selection bias), and survey administration at the point of care whilst the patient was in the ICU (non-response bias and recall bias).

### 2.4 Statistical analysis and sample size

Survey responses were analysed descriptively. Count data were presented as numbers and percentages. For continuous variables, normally distributed data were presented as mean and standard deviation (SD) and non-normally distributed data as median and interquartile range (IQR). The association between respondent characteristics and theoretical willingness to participate in two ICU recovery RCTs was assessed as an odds ratio using univariate logistic regression. A convenience sample of whichever came first between 50 participants for smaller sites or 100 participants for larger sites, or six months of data collection was planned. The estimated total sample size was 300.

## 3 Results

**Fig. 1:**
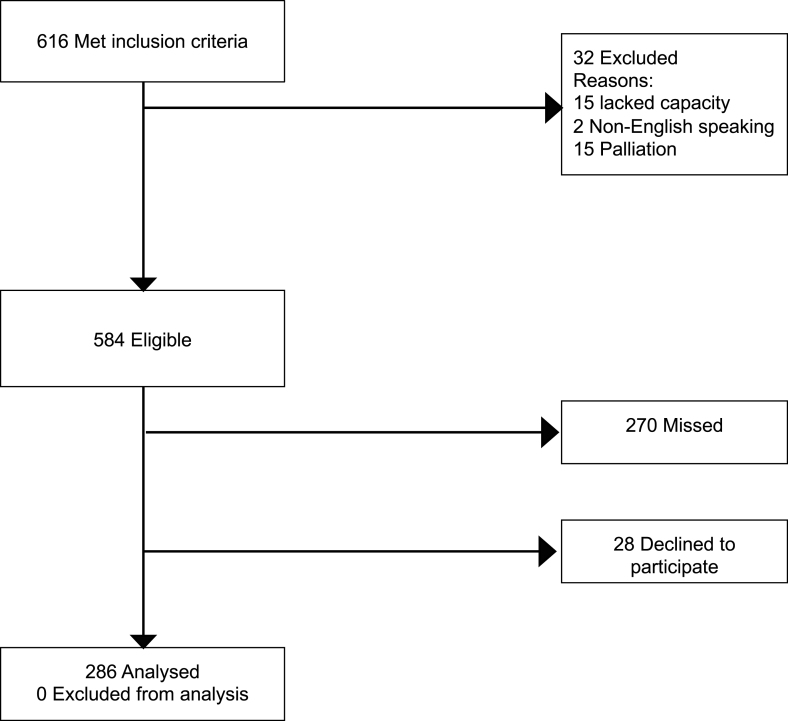
Screened, eligible, and enrolled participants.

Between 23/10/2020 and 4/1/2022 there were a total of 584 eligible patients of whom 314 were approached for participation and 286 (49%) consented to participate and provided survey responses (Fig. 1). The number of responses per site ranged from 17 to 100. There was between site variation in survey start dates (October 2020 to August 2021) and recruitment periods (3–11 months). Missing data was low with one question missing one response and two questions missing two responses.

The mean age of respondents was 62.3 years (SD 14.8) and 178 (62.2%) were male (Table 1). The median ICU length of stay at the time of survey response was 4 days (IQR 3–7), 261 (91.3%) had received vasoactive therapy, 165 (57.7%) mechanical ventilation, and 34 (11.9%) renal replacement therapy. The median EQ5D visual analogue score on the day of survey completion in ICU was 70 (IQR 50–80) and the median estimated expected score increase was 20 (IQR 10–30), with a reported expectation of achieving the improvement in 23 days (IQR 10–33). Respondents reported high levels of concern for all health outcomes with the highest scores being for survival with severe disability and requirement for ongoing care 8 (IQR 3–10) and never being able to return home needing assisted living or nursing home 8 (IQR 1–10), and the lowest scores of 5 (IQR 1–10) for concern about dying.

**Table 1:** Cohort characteristics and reported health concerns.

Characteristic (n = 286)^0^	Value
Age – mean years (SD)	62.3 (14.8)
Male sex – n (%)	178 (62.2)
ICU length of stay – median days (IQR)	4 (3–7)
Unplanned admission - n (%)	187 (65.4)
Admission type – n (%)	
Medical	111 (38.9)
Surgical	175 (61.1)
General surgical	124 (43.4)
Cardiothoracic	43 (15.0)
Trauma	8 (2.8)
Organ support – n (%)	
Mechanical ventilation	165 (57.7)
Vasoactive therapy	261 (91.3)
Renal replacement therapy	34 (11.9)
Haemoglobin – mean g/L	97.8 (18.2)
Health score^+^ - median (IQR), [mean, SD]	
At time of survey	70 (50–80) [67.2, 21.4]
Hoped to be achieved	95 (80–100) [89.6, 14.9]
Change	20 (10–30) [22.4, 19.9]
Patient-reported estimate of time to achieve recovery – median days (IQR)^2^	23 (10–33)
Patient-reported health concerns∗ - median (IQR)	
Surviving but being severely disabled and requiring ongoing care	8 (3–10)
Needing a long time in hospital to recover^1^	7 (3–9)
Requiring repeated trips to hospital after discharge	6 (2–9)
Not being able to return to previous levels of activity^1^	7 (4–9)
Uncontrolled pain^2^	7 (2–10)
Ongoing anxiety and or low mood	6 (3–8)
Never being able to return home, needing assisted living or nursing home	8 (1–10)
Dying	5 (1–10)
Reported willingness to participate in an ICU recovery trial - n (%)	
Iron and EPO for functional recovery	217 (75.9)
Anabolic steroids to address weakness	174 (60.8)

Characteristic (n = 286)^0^	Reported willingness to participate in an ICU recovery RCT Odds ratio (95% CI)^ˆ^	
	Iron & ESA RCT	Anabolic steroids RCT
Age – mean years (SD)	0.99 (0.97–1.01)	0.98 (0.96–0.99)
Male sex – n (%)	1.48 (0.86–2.57)	**1.94** (1.19–3.17)
ICU length of stay – median days (IQR)	1.00 (0.96–1.05)	1.01 (097–1.06)
Unplanned admission - n (%)	1.29 (0.74–2.27)	0.89 (0.54–1.47)
Admission type – n (%)	0.62 (0.35–1.10)	0.91 (0.56–1.49)
Medical		
Surgical		
General surgical		
Cardiothoracic		
Trauma		
Organ support – n (%)		
Mechanical ventilation	1.45 (0.84–2.50)	1.40 (0.87–2.26)
Vasoactive therapy	1.25 (0.50–3.13)	0.71 (0.30–1.71)
Renal replacement therapy	1.26 (0.52–3.03)	1.62 (0.75–3.56)
Haemoglobin – mean g/L	0.99 (0.98–1.01)	1.00 (0.98–1.01)
Health score^+^ - median (IQR)		
At time of survey	1.00 (0.99–1.01)	1.00 (0.99–1.01)
Hoped to be achieved	1.00 (0.99–1.03)	1.01 (0.99–1.02)
Change	1.00 (0.99–1.01)	1.00 (0.99–1.01)
Patient-reported estimate of time to achieve recovery – median days (IQR)^2^	1.00 (0.98–1.02)	0.99 (0.98–1.01)
Patient-reported health concerns∗ - median (IQR)		
Surviving but being severely disabled and requiring ongoing care	**1.11** (1.03–1.19)	1.00 (0.94–1.07)
Needing a long time in hospital to recover^1^	1.06 (0.98–1.15)	1.00 (0.94–1.07)
Requiring repeated trips to hospital after discharge	1.03 (0.95–1.12)	0.99 (0.92–1.06)
Not being able to return to previous levels of activity^1^	**1.10** (1.01–1.19)	0.98 (0.91–1.05)
Uncontrolled pain^2^	**1.08** (1.01–1.17)	0.96 (0.90–1.02)
Ongoing anxiety and or low mood	1.05 (0.97–1.14)	0.97 (0.90–1.04)
Never being able to return home, needing assisted living or nursing home	**1.09** (1.02–1.16)	0.97 (0.92–1.03)
Dying	1.06 (1.00–1.14)	0.99 (0.93–1.05)

^0^ = no missing responses unless otherwise stated, ^1^ = 1 missing response, ^2^ = 2 missing responses. ESA erythropoiesis stimulating agent, SD standard deviation, ICU intensive care unit, RCT randomised clinical trial, IQR interquartile range, ∗higher score indicating greater concern with zero ‘no concern at all’ and 10 ‘extremely concerned’, ^+^Based on EuroQuol visual analogue scale with zero indicating worst health state possible and 100 indicating best health state possible, ^ˆ^Highlighted where P-value<0.05.

A majority of respondents reported theoretical willingness to participate in ICU recovery trials. Male sex was the only participant demographic associated with a difference in reported willingness to participate in an RCT (anabolic steroids). Higher health concerns of being severely disabled requiring ongoing care, not being able to return to previous activity levels, uncontrolled pain and never being able to return home were associated with increased odds of being willing to participate in the iron and ESA RCT (Table 2). Additional qualitative health concerns were reported by 39 participants and most commonly included physical concerns (e.g., sleep), social concerns (e.g., being unable to return to work), and specific disease-orientated concerns (Table 2).

**Table 2:** Other health concerns.

Domain	Additional Concern (n = 39)
Physical	Not sleeping Not sleeping Not being able to attend to personal care Deconditioning Falling Falling Being unable to walk Mobility and independence Chronic pain Shoulder and back pain Dying in hospital
Mental/Cognitive	Unable to stop smoking and alcohol Understanding communication and explanation of interventions Ongoing illicit drug and alcohol use Delirium or dementia Losing memory
Social	Being a burden on family Being a burden to family Being a burden to family Being burden on family Worries about family Worries about family coping Just want to go home Lack of support at home Unable to work Financial stress not being able to work whilst in hospital Not being able to return to work Being unable to return to work Leaving partner in aged care not being about to care for partner Lack of continuity of healthcare Carer responsibilities Hospital disability allowance
Disease-orientated	Scarred of recurrence Cancer Lung cancer Blood malignancy Setting up home dialysis Renal failure Kidney disease Kidney disease Arthritis COVID Heart issues Peripheral vascular disease Having a TIA Failing to recover Progression of other medical conditions Managing other illnesses Medical procedures going wrong

## 4 Discussion

This multicentre survey of 286 critically ill patients has several important findings. Respondents reported generally high levels of health concerns across the eight domains surveyed. Their highest health concerns related to surviving with severe disability and needing ongoing care, and never being able to return home needing assisted living or nursing home. Their lowest health concern was dying. Health priorities are likely to undergo temporal change over the trajectory of critical illness, potentially diminishing death as a concern as ICU survival becomes more likely. Consistent with other studies, these findings demonstrate that ICU survivors are highly concerned about outcomes beyond survival and highlight important considerations for clinicians in evaluating treatment goals related to ICU admission.^7^^,^^8^ For researchers designing studies, these findings suggest that optimising functional recovery is an overarching priority for ICU survivors.

Respondents reported health scores whilst in ICU were only slightly lower than mean Australian 55–64-year-old population EQ-5D health norms of 74.0 (SD 21.1), and the expected improvement substantially higher.^9^ There are limited comparable health scores whilst recovering from critical illness in ICU but could reasonably be expected to be lower and rise less. The causes of the observed and expected discrepancy are uncertain. The responses may reflect limitations of the survey administration in ICU or its predictive validity. However, if replicated, higher health scores may signify the importance placed on health and independence and the mismatch between desired and achieved health outcomes may reflect a previously underreported contributor to post-ICU distress.

Post-intensive care syndrome (PICS) describes a constellation of symptoms that may frequently persist for months or longer and substantially influence reported health concerns.^10^ However, there are limited studies reporting ICU survivors’ expectations for the time course of their recovery. In this survey, respondents anticipated achieving their health improvement in a median of only 23 days, an estimate at odds with prolonged symptoms experienced by many ICU survivors. Similar to the absolute predicted health recovery, an awareness that there may be a mismatch between perceived and likely time required for health recovery may help clinicians in preparing patients expectations post-ICU.

Theoretical willingness to participate in an ICU recovery trial was high. Amongst participant characteristics, only male sex was associated with an increased willingness to participate in an anabolic steroid RCT. Higher concern of surviving with disability requiring ongoing care and not being able to return home were associated with an increased theoretical willingness to participate in an RCT aimed at improving functional recovery. These findings suggest that aligning trial interventions and outcomes with patient priorities may increase participation likelihood.

The study has several limitations. Only half of eligible participants were surveyed so responses may not be representative of all ICU survivors. Health concerns of family members are important but were not collected. Reported responses may differ from actual behaviour and concerns. The findings are not generalisable to ICU patients who lacked the capacity or died in ICU and although consecutive patients were approached, non-responders may report different concerns from responders. The study was undertaken during the COVID-19 pandemic with resultant effects on the overall ICU cohort that may have further implications for generalisability. Finally, factors that may have influenced responses including pre-illness health status, frailty and illness severity were not recorded. However, survey participation was contingent on having received a period of organ support and a majority of patients were unplanned admissions requiring mechanical ventilation.

## 5 Conclusions

Survivors of critical illness surveyed prior to ICU discharge reported high and varied health concerns. The highest concerns related to severe disability requiring ongoing care and not being able to return home. The lowest concern was related to dying. Respondents anticipated that they would complete their recovery over a relatively short timeframe.

## Conflict of interest

The authors declare the following financial interests/personal relationships which may be considered as potential competing interests: Edward Litton is a CC&R Editor If there are other authors, they declare that they have no known competing financial interests or personal relationships that could have appeared to influence the work reported in this paper.

## CRediT authorship contribution statement

EL conceived the study, RAK and EL designed the protocol, all authors contributed to data collection, EL and RAK conducted the initial data analysis with input from all authors, EL wrote the first draft of the manuscript, All authors contributed to subsequent revisions.
